# Prevalence of Sexual Violence and Its Associated Factors Among Pregnant Women in Arba Minch Town, Southern Ethiopia: A Cross‐Sectional Study

**DOI:** 10.1155/jp/5189116

**Published:** 2026-03-13

**Authors:** Arega Abebe Lonsako, Tsehaynew Kasse, Fekadu Abera Kebede, Addisalem Haile

**Affiliations:** ^1^ School of Nursing, College of Medicine and Health Sciences, Arba Minch University, Arba Minch, Ethiopia, amu.edu.et; ^2^ Department of Nursing, College of Health Sciences, Oda Bultum University, Chiro, Ethiopia, obu.edu.et

**Keywords:** Ethiopia, predictors, pregnant women, sexual violence

## Abstract

**Background:**

Sexual violence during pregnancy is a critical global public health concern with severe consequences for maternal and neonatal outcomes, particularly in low‐ and middle‐income countries like Ethiopia. Although previous studies in Ethiopia have primarily focused on general forms of violence, evidence specifically addressing sexual violence during pregnancy remains limited. Therefore, this study is aimed at assessing the prevalence of sexual violence and its associated factors among pregnant women in Arba Minch Town, Southern Ethiopia.

**Method:**

A community‐based cross‐sectional study was conducted among 411 pregnant women from September 15 to October 30, 2024. Participants were selected using a systematic random sampling technique. Data were collected through an interviewer‐administered questionnaire, entered into EpiData Version 4.6, and exported to SPSS Version 26 for analysis. Logistic regression was performed, and variables with *p* < 0.05 were considered statistically significant.

**Result:**

The prevalence of sexual violence was 17% (95% CI: 13.4–20.4). Being a housewife (AOR = 4.0; 95% CI: [1.08, 8.91]), maternal age 15–24 years (AOR = 2.3; 95% CI: [1.08, 5.28]), having a partner with no formal education (AOR = 4.2; 95% CI: [1.84, 9.58]), and partner alcohol consumption (AOR = 2.9; 95% CI: [1.41, 6.37]) were significantly associated with sexual violence.

**Conclusion:**

This study reveals that sexual violence among pregnant women is significant and associated with being a housewife, aged 15–24 years, having an uneducated partner, and a partner who drinks alcohol. Interventions should target at‐risk groups, including young women and housewives, address partner substance use, and promote education and awareness programs to reduce the prevalence and adverse effects of sexual violence in pregnancy.

## 1. Introduction

Sexual violence is a major global public health concern, affecting millions of women worldwide and contributing to increased maternal morbidity, adverse reproductive outcomes, and long‐term psychological distress [1]. The World Health Organization (WHO) defines sexual violence as any act in which a woman is forced into sexual activity against her will through physical coercion, threats, or degrading treatment [2, 3]. Although pregnancy is often viewed as a period of protection and support, evidence indicates that it may also increase women′s vulnerability to different forms of violence, particularly in low‐ and middle‐income countries [4].

Globally, approximately one‐third of women experience sexual violence at some point in their lives, although the magnitude varies widely by region [5]. The burden is particularly high in sub‐Saharan Africa, where nearly one in three women are affected. East Africa reports higher prevalence rates compared with other subregions of the continent [6]. In Ethiopia, the situation is alarming: the lifetime prevalence of sexual violence among women aged 15–49 is estimated at 59%, far exceeding figures reported in many high‐income countries [3, 7].

Sexual violence during pregnancy is associated with several adverse maternal and fetal outcomes, including pregnancy loss, hypertensive disorders, prolonged labor, preterm birth, and low birth weight [8]. Physiological stress responses linked to violence may disrupt the hypothalamic–pituitary–adrenal axis, thereby increasing obstetric risks. In addition to physical complications, survivors often experience long‐term mental health consequences such as depression, anxiety, and posttraumatic stress disorder, with broader implications for families and health systems [9–12].

Although Ethiopia has implemented legal and policy frameworks aimed at preventing gender‐based violence, sexual violence against women remains widespread [13]. Previous studies conducted in different parts of the country have reported varying levels of sexual violence during pregnancy and identified factors such as partner education, cohabitation status, and substance use as important predictors [14, 15]. However, most existing studies have focused on intimate partner violence in general or relied on facility‐based data. Community‐based evidence that specifically addresses sexual violence during pregnancy remains limited. Therefore, this study is aimed at assessing the prevalence of sexual violence and its associated factors among pregnant women residing in Arba Minch Town, Southern Ethiopia.

## 2. Methods and Materials

### 2.1. Study Design, Area, and Period

A community‐based cross‐sectional study was conducted from September 15 to October 30, 2024, in Arba Minch Town, the administrative center of Gamo Zone in Southern Ethiopia. The town is located approximately 454 km south of Addis Ababa and is administratively divided into 11 kebeles. Based on the 2025 population projection by the Ethiopian Central Statistics Agency, the town has an estimated population of 230,270 residents. Health services in the town include one referral hospital, one general hospital, three health centers, 11 health posts, and several private clinics [16].

### 2.2. Population

The source population included all pregnant women residing in Arba Minch Town. The study population involved selected pregnant women who lived in the town during the data collection period.

### 2.3. Eligibility Criteria

All selected pregnant women who were permanent residents of Arba Minch Town were eligible for inclusion. Pregnant women who were seriously ill or unable to respond to the interview were excluded.

### 2.4. Sample Size Determination and Sampling Procedure

The sample size was determined using a single population proportion formula (*n* = (*Z*(*α*/2))^2^ *P*(1 − *P*)/*d* ^2^), assuming a 95% confidence level, a 4% margin of error, and a prevalence of 19.8% obtained from a similar study conducted in Northwest Ethiopia [17]. After adding a 10% allowance for nonresponse, the final sample size was calculated to be 419.

A multistage sampling technique was employed. Initially, five kebeles were selected randomly from the 11 kebeles in the town. The total sample size was then proportionally allocated to each selected kebele based on the estimated number of pregnant women. Study participants were selected using a systematic random sampling technique. The first participant was chosen by lottery, and subsequent participants were selected at regular intervals (every sixth pregnant woman) until the allocated sample size was achieved.

### 2.5. Operational Definitions

#### 2.5.1. Sexual Violence

A pregnant woman was considered to have experienced sexual violence if she reported at least one of the following: being physically forced to have sexual intercourse, engaging in sexual intercourse due to fear of her partner, or being coerced into humiliating or degrading sexual acts [18].

#### 2.5.2. Women′s Autonomy in Healthcare Decision‐Making

Women were classified as autonomous if they made decisions regarding their healthcare independently or jointly with their partner. Those whose healthcare decisions were made solely by their partner or another individual were considered nonautonomous [19].

### 2.6. Variables of the Study

The dependent variable was sexual violence during pregnancy. Independent variables included maternal sociodemographic characteristics, partner‐related factors, and household decision‐making characteristics.

### 2.7. Data Collection Instruments and Procedures

Data were collected using a structured interviewer‐administered questionnaire adapted from WHO guidelines on violence against women and relevant literature [20, 21]. The questionnaire was prepared in English, translated into Amharic, and back‐translated to ensure consistency. A pretest was conducted outside the study area, and necessary modifications were made before the actual data collection. Trained female health extension workers collected the data under close supervision, and written informed consent was obtained from all participants prior to the interview.

### 2.8. Data Processing and Analysis

Data were entered into EpiData Version 4.6 and analyzed using SPSS Version 26. Descriptive statistics were used to summarize the characteristics of the study participants, and results were presented in text, tables, and figures. Bivariable logistic regression was performed to identify candidate variables, and those with *p* < 0.25 were included in the multivariable logistic regression model. Multicollinearity among independent variables was assessed using the variance inflation factor (VIF), and all variables had VIF ≤ 5. Model fitness was evaluated using the Hosmer–Lemeshow goodness‐of‐fit test (*p* = 0.67), indicating an adequate fit. In the multivariable logistic regression analysis, adjusted odds ratios (AORs) with 95% confidence intervals (CIs) were reported, and variables with *p* < 0.05 were considered significantly associated with sexual violence among pregnant women.

## 3. Result

### 3.1. Sociodemographic Characteristic of the Participants

A total of 411 pregnant women participated in the study, yielding a response rate of 98.7%. The mean age was 25.17 years (SD ± 4.3), with 43.3% aged 15–24 years. Most participants were Protestant (63%) and of Gamo ethnicity (60.6%). Nearly half (48.2%) were housewives, and 31.4% had no formal education. Among partners, 44.8% worked in private business, 62.3% consumed alcohol, 33.3% chewed khat, and 12.7% smoked tobacco (Table 1).

**Table 1:** Sociodemographic characteristics of pregnant women in Arba Minch Town, Gamo Zones in Southern Ethiopia Region, 2024 (*n* = 411).

Variables	Categories	Frequency (*n*)	Percentage (%)
Women age in years	15–24	178	43.3
	25–34	140	34.1
	35–49	93	22.6
Religion	Orthodox	77	18.7
	Muslim	46	11.2
	Protestant	259	63
	Catholic	29	7.1
Ethnicity	Gamo	249	60.6
	Gofa	51	12.4
	Others∗	111	27
Occupations	Housewife	198	48.2
	Private business	87	21.2
	Government employee	126	30.7
Educational level	No formal education	129	31.4
	Primary education	124	30.2
	Secondary education	81	19.7
	College and above	77	18.7
Partner occupations	Farmer	79	19.2
	Private business	184	44.8
	Government employee	148	36
Partner educational level	No formal education	100	24.3
	Primary education	85	20.7
	Secondary education	111	27
	College and above	115	28
Partner alcohol consumption	Yes	256	62.3
	No	155	37.7
Partner habit of chat chewing	Yes	137	33.3
	No	274	66.7
Partner habit of smoking tobacco	Yes	52	12.7
	No	359	87.3

*Note:* Others∗ = Wolaita, Amhara, Tigray, and Oromia.

### 3.2. Household Information of Participants

Among the participants, nearly two‐thirds (69.1%) shared decision‐making on how to use their income with their partners, whereas 87% had autonomy in making health‐related decisions. Additionally, three‐quarters (75.9%) made joint decisions with their partners regarding the number of children, and more than two‐thirds (68.6%) made decisions about family visits together with their spouses (Table 2).

**Table 2:** Household related information of pregnant women in Arba Minch Town, Gamo Zones in Southern Ethiopia Region, 2024 (*n* = 411).

Variables	Response	Frequency (*n*)	Percentage (%)
How to use the money earned by her or her partner	Women alone	109	26.5
	Jointly with partner	284	69.1
	Partner alone	18	4.4
Decision on her own health care	Autonomous	361	87.8
	Nonautonomous	50	12.2
Decision on household purchases	Women alone	84	20.4
	Jointly with partner	308	74.9
	Partner alone	19	4.6
Decisions on how many children to have	Women alone	68	16.5
	Jointly with partner	312	75.9
	Partner alone	31	7.5
Decisions to visits the family, friends, or relatives	Women alone	92	22.4
	Jointly with partner	282	68.6
	Partner alone	37	9
Decisions on whether the women should work outside the home	Women alone	99	24.1
	Jointly with partner	245	59.6
	Partner alone	67	16.3

### 3.3. Prevalence of Sexual Violence Among Pregnant Women

**Figure 1:**
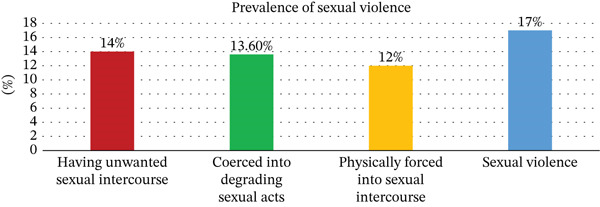
Prevalence of sexual violence among pregnant women in Arba Minch Town, Gamo Zone, Southern Ethiopia Region, 2024.

Of the 411 pregnant women who participated in this study, 70 (17%) reported experiencing sexual violence during pregnancy (95% CI: 13.4%–20.4%). Among them, 59 women (14%) reported having unwanted sexual intercourse out of fear of their partner, 56 women (13.6%) were coerced into degrading sexual acts, and 50 women (12%) were physically forced into sexual intercourse (Figure 1).

### 3.4. Factors Associated With Sexual Violence Among Pregnant Women

In the bivariable logistic regression analysis, mother′s occupations, mother‐age category, decision on the health care, partner′s educational status, partner′s alcohol drinking, partners chat chawing, and partner′s smoking tobacco were factors significantly associated with sexual violence among pregnant women at 95% confidence level with *p* < 0.25, and included in multivariable logistic regression analysis (Table 3).

**Table 3:** Factors associated with sexual violence among pregnant women in Arba Minch Town, Gamo Zone, Southern Ethiopia Region, 2024 (*n* = 411).

Variables	Sexual violence		COR (95% CI)	AOR (95% CI)	*p*
	Yes	No			
*Women occupations*					
Housewife	50 (25.3%)	178 (74.7%)	3.9 (1.90, 8.506)	4.0 (1.80–8.91)	0.001∗
Private business	10 (11.5%)	77 (88.5%)	1.5 (0.5, 3.7)	1.8 (0.66–4.95)	0.249
Government employee	10 (7.9%)	116 (92.1%)	Ref	Ref	
*Women age category*					
15–24	48 (27%)	130 (73%)	2.4 (1.24, 4.97)	2.3 (1.08–5.28)	0.031∗
25–34	10 (7.1%)	130 (92.9%)	0.5 (0.215, 1.25)	0.4 (0.175–1.23)	0.126
35–49	12 (12.9%)	81 (87.1%)	Ref	Ref	
*Decision on women health care*					
Autonomous	58 (16.1%)	303 (83.9%)	Ref	Ref	
Nonautonomous	12 (24%)	38 (76%)	1.6 (0.81, 3.34)	1.1 (0.45–2.65)	0.824
*Partner education*					
No formal education	38 (38%)	62 (62%)	5.7 (2.76, 12.15)	4.2 (1.84–9.58)	0.001∗
Primary school	11 (12.9%)	74 (87.1%)	1.40 (0.57, 3.41)	0.5 (0.19–1.59)	0.281
Secondary school	10 (9%)	101 (91%)	0.9 (0.38–2.30)	0.7 (0.26–1.86)	0.487
College and above	11 (9.6%)	104 (90.4%)	Ref	Ref	
*Partner drinking alcohol*					
Yes	59 (23%)	197 (77%)	3.9 (1.98, 7.72)	2.9 (1.41–6.37)	0.004∗
No	11 (7.1%)	144 (92.9%)	Ref	Ref	
*Partner chat chawing*					
Yes	29 (21.2%)	108 (78.8%)	0.6 (0.38, 1.11)	1.6 (0.83–3.30)	0.152
No	38 (15%)	233 (85%)	Ref	Ref	
*Partner smoking tobacco*					
Yes	13 (25%)	39 (75%)	1.7 (0.88, 3.5)	1.8 (0.71–4.71)	0.203
No	57 (15.9%)	YYY (84.1%)	Ref	Ref	

Abbreviations: AOR, adjusted odd ratio; CI, confidence interval; COR, crude odd ratio; Ref, reference category.

∗*p* < 0.05 (significance).

However, on the multivariable logistic regression analysis being housewives (AOR = 4.0; 95% CI: [1.08, 8.91]), mother age between 15 and 24 (AOR = 2.3; 95% CI: [1.08, 5.28]), no formal education of partner (AOR = 4.2; 95% CI: [1.84, 9.58]), and partner alcohol consumption (*A* *O* *R* = 2.9; 95% CI: [1.41, 6.37]) were factors significantly associated with sexual violence among pregnant women at a 95% confidence level with *p* < 0.05 (Table 3).

## 4. Discussion

This study assessed the prevalence of sexual violence during pregnancy and identified associated factors among pregnant women in Arba Minch Town, Southern Ethiopia. The findings indicate that 17% (95% CI: 13.4, 20.4) of pregnant women experienced sexual violence during the current pregnancy. Being a housewife, younger maternal age (15–24 years), having a partner with no formal education, and partner alcohol consumption were found to be independently associated with sexual violence.

This finding is comparable with studies from Debre Markos, Northwest Ethiopia, the Tigray region, and Tanzania, where the prevalence was 19.4%, 19.8%, 15.5%, and 18.8%, respectively [14, 15, 17, 22]. However, the prevalence in Arba Minch was higher than reports from Harar (3.7%) and Gondar (7.6%) [23, 24]. This difference may be attributed to the timing of the studies, as more recent studies are likely to reflect improved awareness, disclosure, and reporting of sexual violence. It may also be influenced by sociocultural variations that shape women′s willingness to disclose such experiences. In settings where stigma, fear of consequences, or limited support, underreporting remains more common [25, 26].

Conversely, the prevalence in our study was significantly lower than that in the Iran (25.3%) and Pakistan (34.6%) studies [27, 28]. This variation may reflect differences in how sexual violence is defined and measured across studies. Some employ broader definitions that include emotional coercion or nonconsensual marital acts, whereas others capture only acts involving physical force. Differences in data‐collection methods may also influence disclosure. In many settings, more private or self‐administered approaches tend to yield higher reporting of sensitive experiences than face‐to‐face interviews, as they reduce fear, embarrassment, and social desirability bias [29–31].

Regarding occupational status, housewives were four times more likely to experience sexual violence compared with employed women. This finding aligns with studies conducted in Debre Markos and North West Ethiopia [14, 17]. This association may be explained by the fact that women who remain at home often have reduced access to education, income, and social support, which can weaken their autonomy within intimate relationships. In addition, societal expectations that assign women primarily domestic responsibilities may perpetuate power imbalances, making it more difficult for them to oppose or seek assistance for abusive conduct [32].

This study found that pregnant women aged 15–24 years were twice as likely to experience sexual violence compared with their older counterparts, a result consistent with a study conducted in Zambia [33]. This finding may be attributed to the relative vulnerability of younger women, who often enter relationships with limited decision‐making authority and reduced negotiating power. Prevailing social expectations and gender norms may further place younger women at a disadvantage, whereas restricted access to education, legal safeguards, and social support can hinder their ability to prevent, challenge, or leave abusive relationships [33–35].

Pregnant women with partners who had no formal education were four times more likely to be exposed to sexual violence compared to their counterparts. This result aligns with studies conducted in North West Ethiopia and Debre Markos [14, 17]. This may be explained by the fact that partners who have not received formal education may be less aware of women′s legal rights and the principles of gender equity, potentially reinforcing harmful beliefs and behaviors in relationships and creating situations where consent is not fully respected [36].

Finally, partner alcohol use was strongly associated with sexual violence, with women three times more likely to experience abuse if their partners consumed alcohol. This finding aligns with studies conducted in the Tigray region and Debre Markos town [15, 17]. A possible explanation is that alcohol use impairs judgment, lowers self‐control, and heightens aggression, leading intoxicated partners to act impulsively or ignore consent, resulting in abusive behavior. Moreover, alcohol can exacerbate existing conflicts, making violence more likely during the stress of pregnancy [37].

### 4.1. Limitation of the Study

This study is subject to social desirability bias, as the sensitive nature of sexual violence may have influenced participants′ willingness to disclose their experiences, despite assurances of confidentiality. The cross‐sectional design also limits the ability to establish temporal or causal relationships. Future research should consider longitudinal approaches to better capture determinants of sexual violence during pregnancy.

## 5. Conclusion

This study shows that sexual violence among pregnant women remains a pressing public health concern. Key associated factors include being a housewife, younger maternal age (15–24 years), having a partner with no formal education, and having a partner who consumes alcohol. These findings highlight the urgent need for targeted interventions. Clinically, health professionals should integrate routine screening for sexual violence into antenatal care, along with supportive counseling and education that empower women to protect themselves, practice safer sexual behaviors, and seek help when facing violence. At the community level, local authorities in Arba Minch, together with the Ministry of Health, should prioritize awareness and prevention efforts, particularly addressing partner alcohol use and raising community awareness about the risks associated with low educational status and related behaviors.

**Nomenclature**
CSA: Southern Statistical Agency of Ethiopia
DC: data collector
SV: sexual violence
EDHS: Ethiopia Demographic Health Survey
FDRE: Federal Democratic Republic of Ethiopia
WHO: World Health Organization

## Author Contributions

A.A.L., T.K., F.A.K., and A.H. were involved in the conception, design, analysis, interpretation, report, and manuscript writing.

## Funding

No funding was received for this manuscript.

## Disclosure

All authors read and approved the final manuscript.

## Ethics Statement

Ethical approval was obtained from the Institutional Review Board of the Arba Minch University College of Medicine and Health Sciences (IRB/1411/2023). Before data collection, written informed consent was obtained from the study participants, and the right to withdraw from the interview was guaranteed. The privacy and confidentiality of the information obtained from the respondents were kept confidential and anonymous.

## Consent

The authors have nothing to report.

## Conflicts of Interest

The authors declare no conflicts of interest.

## Data Availability

The datasets used and/or analyzed during the current study are available from the corresponding author on reasonable request.
